# Should cigarette pack sizes be capped?

**DOI:** 10.1111/add.14770

**Published:** 2019-08-30

**Authors:** Anna K. M. Blackwell, Ilse Lee, Michelle Scollo, Melanie Wakefield, Marcus R. Munafò, Theresa M. Marteau

**Affiliations:** ^1^ UK Centre for Tobacco and Alcohol Studies School of Psychological Science, University of Bristol Bristol UK; ^2^ Behaviour and Health Research Unit University of Cambridge Cambridge UK; ^3^ Centre for Behavioural Research in Cancer Melbourne Vic Australia

**Keywords:** Cigarette pack size, choice architecture, policy, portion size, public health, tobacco control

## Abstract

**Background:**

Very few countries regulate maximum cigarette pack size. Larger, non‐standard sizes are increasingly being introduced by the tobacco industry. Larger portion sizes increase food consumption; larger cigarette packs may similarly increase tobacco consumption. Here we consider the evidence for legislation to cap cigarette pack size to reduce tobacco‐related harm.

**Aims and analysis:**

We first describe the regulations regarding minimum and maximum pack sizes in the 12 countries that have adopted plain packaging legislation and describe the range of sizes available. We then discuss evidence for two key assumptions that would support capping pack size. First, regarding the causal nature of the relationship between pack size and tobacco consumption, observational evidence suggests that people smoke fewer cigarettes when using smaller packs. Secondly, regarding the causal nature of the relationship between reducing consumption and successful cessation, reductions in number of cigarettes smoked per day are associated with increased cessation attempts and subsequent abstinence. However, more experimental evidence is needed to infer the causal nature of these associations among general populations of smokers.

**Conclusion:**

Cigarette pack size is positively associated with consumption and consumption is negatively associated with cessation. Based on limited evidence of the causal nature of these associations, we hypothesize that government regulations to cap cigarette pack sizes would positively contribute to reducing smoking prevalence.

## Introduction

Many countries have tobacco control legislation that establishes a minimum number of cigarettes that can be included in a single pack. In many countries that have regulated on this the minimum cigarette pack size is 20, e.g. in the United States (Code of Federal Regulations Title 21 Sec. 1140.16) and the European Union member states (EU Tobacco Products Directive, 2014/40/EU). The EU directive imposed a minimum number of cigarettes per pack to increase the upfront cost of cigarettes and thereby make them less affordable for young people 1. By contrast, there is very little regulation regarding maximum pack size, which varies globally between 10 and 50 cigarettes per pack. Packs of 25 were introduced in Australia during the 1970s, and packs of 30, 35, 40 and 50 progressively entered the market over the subsequent two decades 2. In Ireland, pack sizes larger than 20 have grown steadily from 0% of sales in 2009 to 23% in 2018 3. In the United Kingdom, packs of 23 and 24 were introduced following the introduction of plain (standardized) packaging. Learning from these experiences, New Zealand mandated for just two standard pack sizes (20 and 25) as part of its legislation for plain packaging 4.

The availability of pack sizes larger than 20 cigarettes is of particular interest because of growing evidence for the role of portion size in consumption of other products. Consumption of food increases when people are offered larger, compared to smaller, portion sizes, with a Cochrane systematic review finding a small to moderate effect of portion size on food and soft‐drink consumption 5. The review also examined evidence for the effect of portion size on tobacco consumption. Only three studies met the inclusion criteria, all focused on cigarette length, with no studies examining the impact on consumption of cigarette pack size. The dearth of experimental evidence is a concern, because increasing availability of larger pack sizes could undermine improvements to public health achieved through other tobacco control policies.

To date, the success of tobacco control policies in many countries has largely been due to reducing uptake through price‐based interventions rather than promoting cessation, with cessation rates remaining relatively constant over time 6. This challenge emphasizes the need for policies that encourage cessation. Reducing the number of cigarettes per day that smokers consume may be an important precursor to successful cessation attempts, and while increasing prices is probably the most effective strategy, other tobacco control policies have also been important in decreasing consumption 7. Trends in smoking have shown that smokers can and have initiated and maintained reductions in consumption in many countries. For example, in the years when no‐smoking policies were increasingly being adopted in work‐places, smokers were more likely to stop smoking in smoke‐free work‐places compared to those that allowed smoking 8. Reported numbers of cigarettes smoked per day has also declined over time in Australia, the United Kingdom and many other countries (2002–07) 9.

In England, the National Institute for Health and Care Excellence (NICE) guidelines (national evidence‐based health‐care recommendations) encourage smokers to reduce consumption on the basis that it is likely to increase chances of cessation. However, there is some concern that promoting reduction may undermine cessation and resistance to relapse 10. A systematic review of smoking cessation interventions found that cutting down prior to stopping, or stopping abruptly, had comparable cessation rates for smokers intending to stop 11. A subsequent trial found that cutting down to stop smoking was less effective than stopping smoking abruptly 12; however, the authors suggested that advice to reduce smoking may still be worthwhile if it increases engagement with the concept of receiving support. An environmental modification such as capping cigarette pack size has the potential to reduce consumption besides conscious awareness. It therefore presents an opportunity to deliver the benefits of reduced consumption without the smoker developing self‐exempting beliefs about reduced harm through reduction alone. Success has been demonstrated from policies to cap the maximum size, and number permitted in a single sale, of other harmful products. For example, reducing the number of analgesic pills per pack has been beneficial in the prevention of deaths by suicide 13.

This paper aims to build on a recent Cochrane review 5 for which no experimental studies were found of the impact of cigarette pack size on tobacco consumption. In the absence of direct evidence, we have identified existing variation in availability of pack sizes and synthesized the literature relevant to two key assumptions for capping pack size: (i) reducing pack size can reduce consumption; and (ii) reducing consumption can increase cessation. The dearth of experimental studies to support these assumptions does not preclude the threat that increasingly large cigarette pack sizes (> 20) may pose to the success of other tobacco control policies. We contend that the regulatory focus regarding minimum pack size, without due consideration of whether there should be a mandatory maximum pack size, has essentially created a loophole that the tobacco industry can exploit. Based on indirect evidence we propose the hypothesis that Government regulation to cap cigarette packs to 20 cigarettes would contribute to national and global tobacco control policies to reduce smoking prevalence.

## Argument and analysis

### Variation in cigarette pack size regulation and availability across countries

As far as we are aware, only three countries have legislated for the specific number of cigarettes that can be included in a single pack: in both Russia (Federal Law no. 15‐FZ) and Georgia (Legislative Herald of Georgia no. 4059‐RS, 15.12.2010) only pack sizes of 20 are permitted for sale, and in New Zealand pack sizes must be either 20 or 25 (Smoke‐free Environments Regulations 2017). Many other countries have a regulated minimum cigarette pack size, but not specified a maximum. To determine variation in pack size we first identified a sampling frame. For pragmatic reasons we limited our search to countries that had adopted legislation for plain packaging, some of which have seen the introduction of larger packs following these changes 14. We identified a source document to ascertain the relevant countries 15. We then reviewed publicly available legislation and contacted local policymakers or organizations, as well as tobacco researchers, through a targeted approach based on the countries of interest and through a general query sent to the Society for Research on Nicotine and Tobacco (SRNT) members, to identify the minimum, maximum and range of pack sizes available in different countries (Table 1).

**Table 1:** Pack size regulation and availability in countries where plain packaging has been adopted.

Country	Tobacco control legislation	Regulated minimum pack size	Regulated maximum pack size	Available pack sizes	Details of plain packaging legislation (implementation date at retailer level)
Plain packaging regulation adopted and implemented					
Australia	Tobacco Plain Packaging Act 2011	20	NA	20, 21, 22, 23, 25, 26, 30, 35, 40, 50	Tobacco Plain Packaging Act 2011 (December 2012)
France	EU Tobacco Products Directive	20	NA	20, 25, 30, 35, 40^a^	Code of Public Health Order no. 2016–623 (January 2017)
UK	EU Tobacco Products Directive	20	NA	20, 23, 24^b^	Standardized Packaging of Tobacco Products Regulations 2015 (May 2017)
Ireland	EU Tobacco Products Directive	20	NA	20, 23, 24, 25, 27, 28, 29, 30^c^	The Public Health (Standardized Packaging of Tobacco) Act 2015 (September 2018)
Hungary	EU Tobacco Products Directive	20	NA	20 (single pack size guidance)^d^	Decree no. 239/2016 (May 2019, new brands August 2016)
Norway	Act No. 5 (February 2017) Prevention of the Harmful Effects of Tobacco (Tobacco Control Act)	20	NA	20^e^	Act No. 5 Prevention of the Harmful Effects of Tobacco (Tobacco Control Act) (July 2018)
New Zealand	Smoke‐free Environments Regulations 2017	20	25	Only 20 or 25 permitted	Smoke‐free Environments (Tobacco Standardized Packaging) Amendment Act 2016 (June 2018)
Plain packaging regulations adopted but not yet implemented					
Slovenia	EU Tobacco Products Directive	20	NA	20, 21, 22, 23, 37^f^	Parliament passed bill that includes provisions to introduce no‐brand packaging by January 2020
Romania	EU Tobacco Products Directive	20	NA	20^g^	Parliament passed a law that includes provisions to introduce plain‐packaging regulations
Canada	Tobacco and Vaping Products Act (S.C. 1997, c. 13)	20	NA	20, 25^h^	An act was adopted providing ministerial powers to implement plain packaging regulations
Thailand	Tobacco Products Control Act (2017)	20	NA	20^i^	The Tobacco Products Control Act includes provision (Article 38) to introduce plain packaging
Georgia	Tobacco Control 2010	20	20	20	President signed amending law on Tobacco Control 2017 including plain packaging provision

a https://www.maisondesburalistes.fr/Logista/TARIFS_LOGISTA_AU_30_avril_2018.pdf

b Moodie *et al*. 4.

c Information from the National Tobacco Control Office, Ireland (personal communication).

d http://www.fokuszpont.dohanyzasvisszaszoritasa.hu/hu/content/dohanytermekek-szabalyozasa-csomagolas-cimkezes (single packaging with uniform size) and http://nemzetidohany.hu/wp-content/uploads/2018.05.20.-TPD-Plain-Packaging_ODBE-kieg._V7-weboldalra.pdf.

e Little or no known current variation (personal communication with Norwegian Institute of Public Health).

f Information from Ministry of Finance (personal communication with National Institute of Public Health Slovenia).

g No known current variation (personal communication with researchers at the University of Medicine, Pharmacy, Sciences and Technology of Tirgu Mures, Romania).

h Information from Ontario Tobacco Research Unit, Canada and the Tobacco Control Directorate, Health Canada (personal communication).

i No known current variation (personal communication with the Tobacco Control Research and Knowledge Management Center, Bangkok, Thailand).

From November 2018, plain packaging has been implemented in seven countries 15: Australia, France, United Kingdom, Ireland, Hungary, Norway and New Zealand. Legislation that includes provision for plain packaging has been adopted but not yet implemented in an additional five countries: Slovenia, Romania, Canada, Thailand and Georgia. In all these 12 countries, the minimum pack size available for sale is 20 cigarettes. This is also the largest size available in Georgia, where there is legislation for a single size, Hungary, Norway, Romania and Thailand. In New Zealand, where there is legislation for two standard sizes, and Canada, only pack sizes of 20 and 25 are available. In the other five countries, a larger selection of sizes is available, with the widest range evident in Australia.

### The relationship between pack size and consumption

First, we review evidence for the assumption that cigarette pack size is associated with level of tobacco consumption, which is fundamental for determining its importance as a target for tobacco control. Smokers who use packs containing more cigarettes tend to smoke more cigarettes per day 16. It is possible that this association is the result of self‐selection. For example, heavier smokers may buy larger pack sizes and lighter smokers and those wanting to cut down may buy smaller pack sizes. An experimental study demonstrated that some smokers used small packs as a means of self‐control, and that packs with greater numbers of cigarettes need to be considerably cheaper per stick in order to encourage a switch to larger pack sizes 17. Analysis of data from a large national survey suggested that smokers regulate their consumption according to available pack sizes 18.

Price is a key motivator for manipulating pack size 19: smaller packs have a lower cost per package, whereas larger packs have a lower cost per stick. The former could be used to attract consumers from competitor brands, whereas the latter may appeal to brand committed, price‐sensitive or heavier smokers 19. Box 1 provides further discussion of industry manipulation of pack size as a price‐related marketing strategy. Smokers in clinical trials provided with free cigarettes increase their consumption 20, which could reflect affordability or availability. Data from the International Tobacco Control study 9 provide a useful comparison of tobacco consumption among countries with different pack size availability. The findings show a tendency for smokers in Australia—where pack sizes from 20 to 50 cigarettes have been available for many decades—to smoke more cigarettes per day than smokers in the United Kingdom where, until recently, cigarettes have been sold in packs of 20 and fewer. However, this may be at least partially explained by lower affordability of tobacco in the United Kingdom at the time of the survey 9.

Box 1 **Proliferation of pack size as a price‐related marketing strategy**

Cigarette pack size proliferation—the increasing availability of larger and non‐standard sizes—has become an important form of price‐related marketing used by the tobacco industry. It has emerged in countries where other forms of promotion are restricted and can undermine the effectiveness of tax increases. For example, Australia experienced a proliferation of new pack sizes following the imposition of a 25% increase in tobacco tax in late April 2010, with packs in sizes of 21, 22 and 26 appearing to provide bonus cigarettes and packs of 23 providing a lower price than the previously most popular 25s 46.

More recently, double‐pack bundles of 30s or 40s are being marketed in on‐line Australian supermarkets as 60s or 80s, which provide a substantially lower upfront purchase cost than buying in large cartons (e.g. in 2018, a carton of 200 Peter Jackson 20s cost $270, compared to $75 for a 60s bundle). In the United Kingdom, non‐standard pack sizes have also been introduced following the implementation of plain packaging 14. Prohibiting sales in the form of multiple packs, bundles or cartons would be a useful additional strategy to reduce the availability and affordability of cigarettes.

The use of pack size proliferation to differentially promote cigarette products and confuse price signals for consumers is an additional, related concern to the increasing maximum size. The optimal pack size must be considered in the context of growing evidence for the role of portion size in consumption of other products. Such effects are exacerbated by price promotions that incentivize the purchase of larger portions 47. Tobacco control policies should address overall affordability (e.g. by mandating a minimum size) portion size (e.g. by mandating a maximum size) and pack size marketing (e.g. by mandating a range of sizes between the minimum and maximum). Standardizing specific pack sizes would greatly reduce the capacity of tobacco companies to engage in price‐related promotion. If the association between pack size and consumption is assumed to be causal, then there is a strong case for standardizing a single pack size, allowing for no pack sizes larger than the common minimum size of 20 cigarettes.

Evidence to support a causal link between cigarette pack size and consumption, with larger packs leading smokers to smoke more cigarettes, comes from tobacco industry documents 19. Industry analysts supported by modelling suggested that making cigarettes available in packs of 25, rather than 20, would help to reverse declines in volume by encouraging smokers to increase their daily consumption. Furthermore, the introduction of large packs in Australia (25, 30, 40 and 50) were credited as early as 1990 with maintaining industry sales volumes despite declining prevalence and widespread introduction of smoking bans in Australian work‐places 21. However, as price is an important driver for consumption, the observed increase in sales volumes in Australia could have been a response to the substantially lower prices per stick of cigarettes sold in larger packs rather than the bulk packaging *per se*.

Perhaps the strongest support for the idea that larger pack sizes increase consumption comes from an analysis of smoking habits after packs of 25 were test‐marketed in four areas of the United States in the 1980s. Kozlowski 22 noted that more Canadians, where packs were commonly sold containing 25 cigarettes, reported smoking 25 cigarettes per day, and that as the percentage of packs of 25 on the cigarette market in four regions of the United States increased, so did the percentage of smokers reporting smoking 25 cigarettes per day. However, industry predictions were not always borne out, and introducing 25 packs in the United States had limited success, which was attributed to many smokers’ desire to cut down their consumption 19. Further experimental evidence is required to understand more clearly the nature of the relationship between pack size and consumption—for example, examining the impact on consumption of smokers using smaller packs than they usually do—while recognizing that motivation to reduce or stop smoking and price are important mediating factors.

### The relationship between consumption and cessation

Efforts to cap cigarette pack size should only be a policy target if the association with tobacco consumption is shown to be causal and there is an expected health benefit. There are some small direct health benefits of reducing consumption, but the long‐term effects are not clear 23, and smoking even one cigarette per day carries a much greater risk of developing coronary heart disease and stroke than might be expected 24. Other studies have found no evidence that smoking reduction reduces mortality 25, 26. However, reducing consumption might reduce morbidity and mortality if it increases successful smoking cessation 27. Several theoretical reasons support this assumption, including the benefits of decreased nicotine dependence 28 and increased self‐efficacy 29 for successful cessation. Smoking fewer cigarettes may also reduce exposure to smoking‐related cues, which may boost cessation rates and reduce relapse 30, 31. Below, we discuss evidence for the second key assumption for capping pack size: that reducing consumption of cigarettes per day is associated with increased smoking cessation.

Indirect evidence from population surveys suggest a possible beneficial effect of reducing consumption on increasing the chances of cessation. Low‐intensity smokers (e.g. < 10 cigarettes per day) are more likely to try to stop smoking and succeed, compared to heavy smokers (e.g. > 25 cigarettes per day) 32, 33, 34, 35, and reducing consumption has been reported by smokers as a common cessation strategy 35. Hyland and colleagues 10 analysed trends among participants in the surveys evaluating the US COMMI(T) Trial and found an increase in cessation rates among those who reduced their daily cigarette consumption by 50% or more. However, they noted that very few smokers were able to maintain this level, they did not have information on why people chose to reduce and it is not clear whether they had additional support [e.g. nicotine replacement therapy (NRT)]. Observational evidence prevents causal conclusions from being made about the association found and those smoking less may have been less dependent and more motivated to stop smoking.

Stronger evidence for the causal association between reducing consumption and smoking cessation comes from intervention trials. A systematic review of randomized controlled trials of smoking reduction interventions found that they increased smoking cessation (≥ 6 months) for smokers not motivated to stop smoking 36. All the studies included in the review provided pharmacological support for reduction (e.g. NRT), except one that provided behavioural support alone 37 and demonstrated limited evidence for its effectiveness. Klemperer and Hughes 38 conducted a qualitative review and found that increased reduction in cigarettes per day was associated with increased cessation: the intervention trials included in their review that reported effect sizes suggested that a reduction of 25% should increase the odds of cessation by 75–100%. The authors suggested that this dose–response relationship demonstrated the importance of reduction itself, but noted that the studies all included covariates, which makes it difficult to discount the role of NRT or motivation. In an analysis of longitudinal data*,* Klemperer and colleagues 39 found that participants who did not intend to stop smoking but who reported reduced consumption were more likely to attempt to stop, which increased according to the size and period of reduction. However, it remains difficult to exclude the role of participants’ reasons to reduce, which the authors noted could have been increased through repeated study assessments. Begh and colleagues 27 also highlighted the common lack of long‐term abstinence measures in reduction interventions and suggested that studies should follow recommendations in this area 40. There is currently insufficient experimental evidence that reducing smoking alone improves subsequent cessation without additional support, which is needed to understand the extent to which the association is causal.

### Potential population impact of capping cigarette pack size

Mendelian randomization studies use genetic variants associated with an exposure of interest (e.g. smoking) in an instrumental variable analysis to provide evidence about causal relationships. There is evidence that, among ever smokers, carriers of the rs16969968‐rs1051730 risk allele (associated with an increase in heaviness of smoking of one cigarette per day) are more likely to be current smokers than former smokers [odds ratio (OR) per allele = 1.09, 95% confidence interval (CI) = 1.07–1.11] 41. This suggests that heaviness of smoking is causally related to smoking cessation, indicating that for every additional cigarette smoked per day the odds of being a smoker (rather than a former smoker) increases by 9%, and provides a basis for believing that reducing heaviness of smoking should increase rates of cessation. If we conservatively assume a 5% increase in the odds of cessation for each cigarette fewer smoked per day, reductions of one, three or five cigarettes per day would reduce smoking prevalence by 0.1, 0.4 or 0.7%, respectively, over 1 year, or 0.6, 2.0 or 3.5% if maintained over 5 years (based on the numbers of additional ex‐smokers outlined in Table 2). The population‐level impact of measures that successfully reduce cigarette consumption will depend on the size of the population, the prevalence of smoking and the extent of reduction achieved, illustrated in Table 2.

**Table 2:** Expected impact of reductions in cigarettes smoked per day on additional ex‐smokers over 1 year.

		Number of additional ex‐smokers^c^		
Adult population^a^	Smoking prevalence^b^	1 cigarette reduction	3 cigarette reduction	5 cigarette reduction
Thailand (54 500 000)	21%	14 306	45 100	79 051
France (51 700 000)	34%	21 973	69 268	121 412
Canada (29 000 000)	13%	4713	14 856	26 040
Slovenia (1 700 000)	24%	510	1608	2818

a Based on approximately 80% of the whole population size provided by the World Health Organization 45 country profiles.

b Current adult tobacco, or cigarette use (depending on data availability) provided by the World Health Organization 45 country profiles.

c Calculations assume an effect of cigarette reduction on likelihood of stopping smoking equivalent to odds ratio (OR) 1.05 per cigarettes per day reduction, and a base rate of cessation in the population of 5% among those who attempt to stop smoking (assumed to be 50%) (illustrated using countries that have adopted plain packaging with large or small populations and high or low smoking prevalence).

On the basis of these assumptions and estimated effects of reducing consumption, if reduced pack size is demonstrated to reduce consumption, then capping pack size could offer comparable reductions in smoking prevalence to other tobacco control strategies, such as active quit lines (0.8%), health‐care provider interventions (1.6%) and rotating graphic health warnings (5%) 42. Even small reductions in smoking would have an important impact on rates of smoking cessation.

### Strengths and limitations

This article is the first attempt, to our knowledge, to draw attention to the potential consequences of the availability of larger (> 20) cigarette pack sizes and bring together evidence to support decision‐making regarding pack size regulation. We have highlighted the availability of different pack sizes in countries that have adopted plain packaging which, despite regulations for minimum size, vary widely in the range and maximum size on offer. This is particularly concerning given the dose–response relationship between level of tobacco consumption, including cigarettes per day and negative health outcomes 43. However, it is important to recognize a number of limitations. First, we only sampled a small number of high‐ and middle‐income countries, which may under‐ or overstate the global variation in pack size. Secondly, the lack of experimental studies reported in a Cochrane review 5, and the variation in the type of literature we have synthesized here, precludes a robust analysis of the overall strength and quality of the evidence presented. We have instead identified and triangulated evidence from a range of different sources, including industry documents and analyses, population surveys, intervention trials and Mendelian randomization analyses. These converge regarding the assumptions that consumption increases with pack size, while cessation increases with reduced consumption. We emphasize the need for experimental research in this area to address the causal nature of these relationships.

## Conclusions

Cigarette pack size is increasing in several markets around the world, and the current limited regulatory attention to pack size is being exploited by industry for market gain. Countries that have introduced tobacco plain packaging have seen a proliferation of non‐standard pack sizes, which maintain interest in tobacco products, increase affordability of products (either per pack or per stick) and confuse price signals after tax increases 44. Policymakers should consider optimizing pack size regulation to reduce available pack size, affordability and scope for price‐related marketing. A pragmatic approach would be to cap the total number of cigarettes in a pack at 20, which many countries have adopted as a minimum size. This paper has reviewed evidence for the two key assumptions for capping cigarette pack size. Existing observational evidence suggests that greater cigarette pack size is associated with higher cigarette consumption, which suggests that capping pack size could reduce consumption. Evidence from population surveys and intervention trials shows that reducing consumption can facilitate smoking cessation. Experimental evidence is needed to infer the causal nature of the observed associations between pack size and consumption relative to affordability, motivation to stop smoking and nicotine dependence. Such evidence is needed to test our proposed hypothesis that Government regulation to cap cigarette packs to 20 cigarettes would contribute to national and global tobacco control policies to reduce smoking prevalence.

### Declaration of interests

None.

## References

[1] European Commission . 10 key changes for tobacco products sold in the EU: European Commission Press Release Database; 2016 Available at: http://europa.eu/rapid/press-release_IP-16-1762_en.htm (accessed 4 June 2019).

[2] Scollo M. , Bayly M. The price of tobacco products in Australia In: Scollo M. M., Winstanley M. H., editors. Tobacco in Australia: Facts and Issues. Melbourne, Australia: Cancer Council Victoria; 2016 Available at: http://www.tobaccoinaustralia.org.au/chapter-13-taxation/13-3-the-price-of-tobacco-products-in-australia (accessed 4 February 2019).

[3] KildareStreet . Department of Finance: Excise Duties—Written answers, Tuesday, 26 February 2019 (Paschal Donohoe): KildareStreet.com; 2019 Available at: https://www.kildarestreet.com/wrans/?id=2019-02-26a.292 (accessed 28 Februrary 2019).

[4] Moodie C. , Hoek J. , Scheffels J. , Gallopel‐Morvan K. , Lindorff K. Plain packaging: legislative differences in Australia, France, the UK, New Zealand and Norway, and options for strengthening regulations. Tob Control 2018; 10.1136/tobaccocontrol-2018-054483. 30068563

[5] Hollands G. J. , Shemilt I. , Marteau T. M. , Jebb S. A. , Lewis H. B. , Wei Y. *et al* Portion, package or tableware size for changing selection and consumption of food, alcohol and tobacco. Cochrane Database Syst Rev 2015; 9: CD011045. 10.1002/14651858.CD011045.pub2 PMC4579823 26368271

[6] Royal College of Physicians Nicotine Without Smoke: Tobacco Harm Reduction. London, UK: RCP; 2016 Available at: https://www.rcplondon.ac.uk/projects/outputs/nicotine-without-smoke-tobacco-harm-reduction-0 (accessed 20 July 2018).

[7] Levy D. T. , Chaloupka F. , Gitchell J. The effects of tobacco control policies on smoking rates: a tobacco control scorecard. J Public Health Manag Pract 2004; 10: 338–353. 15235381 10.1097/00124784-200407000-00011

[8] International Agency for Research on Cancer (IARC). IARC Handbooks of Cancer Prevention, Tobacco Control, vol. 13: Evaluating the effectiveness of smoke‐free policies. Lyon. France: IARC, World Health Organization; 2009 Available at: https://publications.iarc.fr/_publications/media/download/4002/3e50217ae9fd2c82fbf15d26b1718757b93947b4.pdf (accessed 1 October 2018).

[9] Yong H. H. , Borland R. , Thrasher J. , Thompson M. E. Stability of cigarette consumption over time among continuing smokers: a latent growth curve analysis. Nicotine Tob Res 2012; 14: 531–539. 22311963 10.1093/ntr/ntr242 PMC3337535

[10] Hyland A. , Levy D. T. , Rezaishiraz H. , Hughes J. R. , Bauer J. E. , Giovino G. A. *et al* Reduction in amount smoked predicts future cessation. Psychol Addict Behav 2005; 19: 221–225. 16011395 10.1037/0893-164X.19.2.221

[11] Lindson‐Hawley N. , Aveyard P. , Hughes J. R. Reduction versus abrupt cessation in smokers who want to quit. Cochrane Database Syst Rev 2012; 11: CD008033. 23152252 10.1002/14651858.CD008033.pub3

[12] Lindson‐Hawley N. , Banting M. , West R. , Michie S. , Shinkins B. , Aveyard P. Gradual versus abrupt smoking cessation: a randomized, controlled noninferiority TrialGradual versus abrupt smoking cessation. Ann Intern Med 2016; 164: 585–592. 26975007 10.7326/M14-2805

[13] Zalsman G. , Hawton K. , Wasserman D. , van Heeringen K. , Arensman E. , Sarchiapone M. *et al* Suicide prevention strategies revisited: 10‐year systematic review. Lancet Psychiatry 2016; 3: 646–659. 27289303 10.1016/S2215-0366(16)30030-X

[14] Moodie C. , Angus K. , Mitchell D. , Critchlow N. How tobacco companies in the United Kingdom prepared for, and responded to, standardised packaging of cigarettes and rolling tobacco. Tob Control 2018; 27: e85–e92. 29321273 10.1136/tobaccocontrol-2017-054011

[15] Campaign for Tobacco Free Kids . Global Issues: Plain Packaging. Washington, DC: Campaign for Tobacco Free Kids; 2018 Available at: https://www.tobaccofreekids.org/what-we-do/global/plain-packaging (accessed 1 November 2018).

[16] Hill D. J. , White V. M. , Scollo M. M. Smoking behaviours of Australian adults in 1995: trends and concerns. Med J Aust 1998; 168: 209–213. 9539898 10.5694/j.1326-5377.1998.tb140132.x

[17] Marti J. , Sindelar J. Smaller cigarette pack as a commitment to smoke less? Insights from behavioral economics. PLOS ONE 2015; 10: e0137520. 26356844 10.1371/journal.pone.0137520 PMC4565702

[18] Farrell L. , Fry T. , Harris M. A pack a day for 20 years: smoking and cigarette pack sizes. Appl Econ 2011; 43: 2833–2842.

[19] Persoskie A. , Donaldson E. A. , Ryant C. How tobacco companies have used package quantity for consumer targeting. Tob Control 2018; 28: 365–373. 10.1136/tobaccocontrol-2017-053993 29853560

[20] Shiffman S. , Scholl S. M. Three approaches to quantifying cigarette consumption: data from nondaily smokers. Psychol Addict Behav 2018; 32: 249–254. 29369671 10.1037/adb0000346

[21] Scollo M. Chapter 2: Trends in tobacco consumption In: Scollo MM and Winstanley MH, [editors]. Tobacco in Australia: Facts and issues. Melbourne, Australia: Cancer Council Victoria; 2019 Available at: https://www.tobaccoinaustralia.org.au/chapter-2-consumption (accessed 2 April 2019).

[22] Kozlowski L. T. Pack size, reported cigarette smoking rates, and public health. Am J Public Health 1986; 76: 1337–1338. 3766833 10.2105/ajph.76.11.1337 PMC1646752

[23] Pisinger C. , Godtfredsen N. S. Is there a health benefit of reduced tobacco consumption? A systematic review. Nicotine Tob Res 2007; 9: 631–646. 17558820 10.1080/14622200701365327

[24] Hackshaw A. , Morris J. K. , Boniface S. , Tang J. L. , Milenkovic D. Low cigarette consumption and risk of coronary heart disease and stroke: meta‐analysis of 141 cohort studies in 55 study reports. BMJ 2018; 360: j5855. 29367388 10.1136/bmj.j5855 PMC5781309

[25] Tverdal A. , Bjartveit K. Health consequences of reduced daily cigarette consumption. Tob Control 2006; 15: 472–480. 17130377 10.1136/tc.2006.016246 PMC2563668

[26] Hart C. , Gruer L. , Bauld L. Does smoking reduction in midlife reduce mortality risk? Results of 2 long‐term prospective cohort studies of men and women in Scotland. Am J Epidemiol 2013; 178: 770–779. 23825165 10.1093/aje/kwt038 PMC3755643

[27] Begh R. , Lindson‐Hawley N. , Aveyard P. Does reduced smoking if you can't stop make any difference? BMC Med 2015; 13: 257. 26456865 10.1186/s12916-015-0505-2 PMC4601132

[28] Vangeli E. , Stapleton J. , Smit E. S. , Borland R. , West R. Predictors of attempts to stop smoking and their success in adult general population samples: a systematic review. Addiction 2011; 106: 2110–2121. 21752135 10.1111/j.1360-0443.2011.03565.x

[29] Smit E. S. , Hoving C. , Schelleman‐Offermans K. , West R. , de Vries H. Predictors of successful and unsuccessful quit attempts among smokers motivated to quit. Addict Behav 2014; 39: 1318–1324. 24837754 10.1016/j.addbeh.2014.04.017

[30] Zhou X. , Nonnemaker J. , Sherrill B. , Gilsenan A. W. , Coste F. , West R. Attempts to quit smoking and relapse: factors associated with success or failure from the ATTEMPT cohort study. Addict Behav 2009; 34: 365–373. 19097706 10.1016/j.addbeh.2008.11.013

[31] West R. The multiple facets of cigarette addiction and what they mean for encouraging and helping smokers to stop. J Chron Obstruct Pulmon Dis 2009; 6: 277–283. 10.1080/15412550903049181 19811387

[32] Jarvis M. J. Patterns and predictors of smoking cessation in the general population In: Bolliger C. T., Fagerstrom K. O., editors. The Tobacco Empire, Vol. 28 Basel, Karger; 1997, pp. 151–164.

[33] Zhu S. H. , Sun J. , Hawkins S. , Pierce J. , Cummins S. A population study of low‐rate smokers: quitting history and instability over time. Health Psychol 2003; 22: 245–252. 12790251 10.1037/0278-6133.22.3.245

[34] Borland R. , Yong H. , O'Connor R. , Hyland A. , Thompson M. The reliability and predictive validity of the heaviness of smoking index and its two components: findings from the international tobacco control four‐country study. Nicotine Tob Res 2010; 12: S45–S50. 20889480 10.1093/ntr/ntq038 PMC3307335

[35] Leatherdale S. , Shields M. Smoking cessation: intentions, attempts and techniques. Health Rep 2009; 20: 31–39. 19813437

[36] Asfar T. , Ebbert J. O. , Klesges R. C. , Relyea G. E. Do smoking reduction interventions promote cessation in smokers not ready to quit? Addict Behav 2011; 36: 764–768. 21420791 10.1016/j.addbeh.2011.02.003 PMC3081955

[37] Glasgow R. E. , Gaglio B. , Estabrooks P. A. , Marcus A. C. , Ritzwoller D. P. , Smith T. L. *et al* Long‐term results of a smoking reduction program. Med Care 2009; 47: 115–120. 19106739 10.1097/MLR.0b013e31817e18d1

[38] Klemperer E. M. , Hughes J. R. Does the magnitude of reduction in cigarettes per day predict smoking cessation? A qualitative review. Nicotine Tob Res 2015; 18: 88–92. 25744970 10.1093/ntr/ntv058 PMC5009415

[39] Klemperer E. M. , Hughes J. R. , Naud S. Reduction in cigarettes per day prospectively predicts making a quit Attempt: a fine‐grained secondary analysis of a natural history study. Nicotine Tob Res 2018; 21: 648–654. 10.1093/ntr/nty056 PMC6468134 29579250

[40] Hughes J. R. , Keely J. P. , Niaura R. S. , Ossip‐Klein D. J. , Richmond R. L. , Swan G. E. Measures of abstinence in clinical trials: issues and recommendations. Nicotine Tob Res 2003; 5: 13–25. 12745503

[41] Taylor A. , Munafò M. CARTA consortium. Does mortality from smoking have implications for future Mendelian randomization studies. Int J Epidemiol 2014; 43: 1483–1486. 25125581 10.1093/ije/dyu151 PMC4190520

[42] Levy D. T. , Tam J. , Kuo C. , Fong G. T. , Chaloupka F. The impact of implementing tobacco control policies: the 2017 tobacco control policy scorecard. J Public Health Manag Pract 2018; 24: 448–457. 29346189 10.1097/PHH.0000000000000780 PMC6050159

[43] US Department of Health and Human Services . The Health Consequences of Smoking: A Report of the Surgeon General. Atlanta, GA: Department of Health and Human Services, Centers for Disease Control and Prevention, National Center for Chronic Disease Prevention and Health Promotion, Office on Smoking and Health; 2004.

[44] Scollo M. , Bayly M. , White S. , Lindorff K. , Wakefield M. Tobacco product developments in the Australian market in the 4 years following plain packaging. Tob Control 2018; 27: 580–584. 28993520 10.1136/tobaccocontrol-2017-053912

[45] World Health Organization (WHO) . WHO Report on the Global Tobacco Epidemic, 2017: Monitoring Tobacco Use and Prevention Policies. Geneva, Switzerland: World Health Organization; 2017 Available at: https://www.who.int/tobacco/global_report/2017/en/ (accessed 1 Februrary 2019).

[46] Scollo M. , Occleston J. , Bayly M. , Lindorff K. , Wakefield M. Tobacco product developments coinciding with the implementation of plain packaging in Australia. Tob Control 2015; 24: e116–e122. 24789601 10.1136/tobaccocontrol-2013-051509

[47] Marteau T. M. , Hollands G. J. , Shemilt I. , Jebb S. A. Downsizing: policy options to reduce portion sizes to help tackle obesity. BMJ 2015; 351; 10.1136/bmj.h5863. 26630968

